# Rational peptide design for inhibition of the KIX–MLL interaction

**DOI:** 10.1038/s41598-023-32848-2

**Published:** 2023-04-18

**Authors:** Nao Sato, Shunji Suetaka, Yuuki Hayashi, Munehito Arai

**Affiliations:** 1 Department of Life Sciences, Graduate School of Arts and Sciences, The University of Tokyo, 3-8-1 Komaba, Meguro, Tokyo, 153-8902 Japan; 2 Environmental Science Center, The University of Tokyo, 7-3-1 Hongo, Bunkyo, Tokyo, 113-0033 Japan; 3 Department of Physics, Graduate School of Science, The University of Tokyo, 3-8-1 Komaba, Meguro, Tokyo, 153-8902 Japan

**Keywords:** Protein design, Protein design, Intrinsically disordered proteins, Protein folding

## Abstract

The kinase-inducible domain interacting (KIX) domain is an integral part of the general transcriptional coactivator CREB-binding protein, and has been associated with leukemia, cancer, and various viral diseases. Hence, the KIX domain has attracted considerable attention in drug discovery and development. Here, we rationally designed a KIX inhibitor using a peptide fragment corresponding to the transactivation domain (TAD) of the transcriptional activator, mixed-lineage leukemia protein (MLL). We performed theoretical saturation mutagenesis using the Rosetta software to search for mutants expected to bind KIX more tightly than the wild-type MLL TAD. Mutant peptides with higher helical propensities were selected for experimental characterization. We found that the T2857W mutant of the MLL TAD peptide had the highest binding affinity for KIX compared to the other 12 peptides designed in this study. Moreover, the peptide had a high inhibitory effect on the KIX–MLL interaction with a half-maximal inhibitory concentration close to the dissociation constant for this interaction. To our knowledge, this peptide has the highest affinity for KIX among all previously reported inhibitors that target the MLL site of KIX. Thus, our approach may be useful for rationally developing helical peptides that inhibit protein–protein interactions implicated in the progression of various diseases.

## Introduction

Protein–protein interactions (PPIs) are involved in many biological processes including transcription, signal transduction, and apoptosis, and are implicated in various diseases. The inhibition of PPIs is one of the most promising approaches for the development of drugs that can be used to treat diseases involving PPIs^1^; however, PPI inhibition is difficult when using conventional small molecule compounds, because the binding surface in PPIs is generally large and shallow, and without well-defined binding pockets^2^. Recently, peptides have attracted a considerable amount of attention as a potential drug modality, because they are able to mimic short segments of proteins involving PPIs and are estimated to be involved in 15–40% of PPIs in a cell^3^. In addition, 62% of protein complexes registered in the Protein Data Bank (PDB) are known to have helical interfaces^4^. Thus, the development of PPI inhibitors using peptides, specifically helical peptides, is an attractive strategy in drug discovery^5,6^.

**Figure 1:**
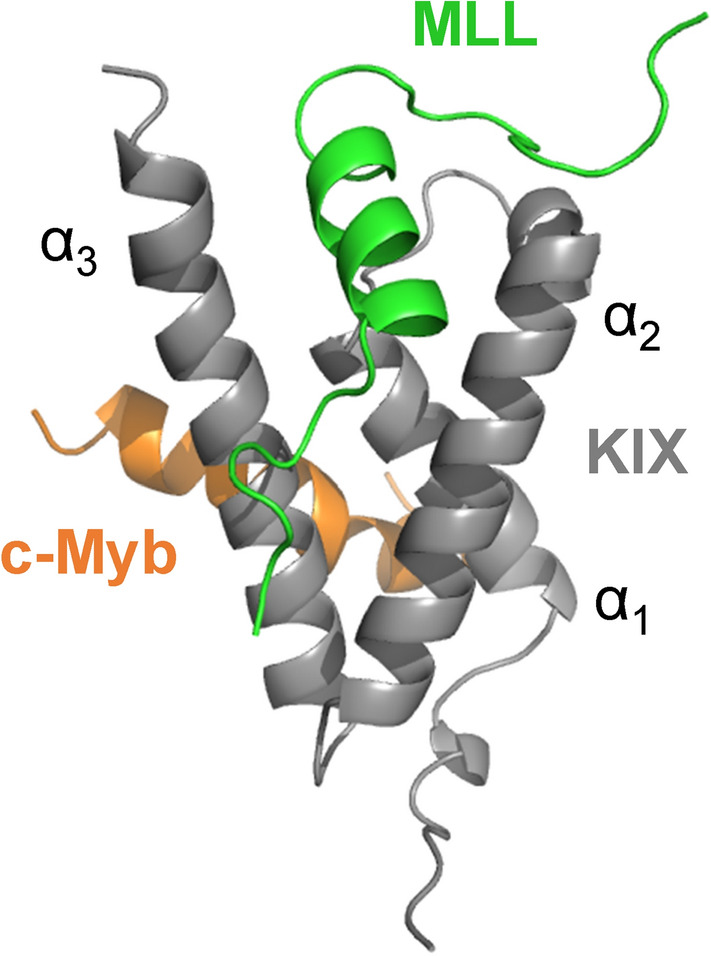
Structure of the KIX:MLL:c-Myb ternary complex. The c-Myb and MLL sites are located at the hydrophobic grooves between helices α_1_ and α_3_ and between helices α_2_ and α_3_ of KIX, respectively (PDB ID: 2AGH). The figure was drawn using PyMOL Molecular Graphics System, Version 2.4.0 Schrödinger, LLC (https://pymol.org).

PPIs frequently occur between transcriptional coactivators and transcriptional activators. The CREB-binding protein (CBP) is a well-known transcriptional coactivator and its kinase-inducible domain interacting (KIX) domain is known to bind various transcriptional activators^7–10^. KIX is a small globular domain composed of three α-helices and two 3_10_-helices^11^. It has two binding sites, the “c-Myb site” and “MLL site”, for transcriptional activators, which bind the transactivation domains (TADs) of the transcriptional activators, c-Myb and mixed-lineage leukemia protein (MLL), respectively (Fig. 1)^11–15^. It has also been reported that the MLL site binds the TADs of various transcriptional activators, including MLL, p53^16^, FOXO3a^17^, HIV-1 Tat^18^, HTLV-1 Tax^19^, and HTLV-1 HBZ^20^. These PPIs have been implicated in serious diseases such as leukemia, cancer, and acquired immunodeficiency syndrome. Therefore, peptide inhibitors that tightly bind the MLL site of KIX may inhibit interactions with these transcriptional activators and have potential applications as therapeutic agents for various diseases.

Previous studies have reported several inhibitors that bind the MLL site of KIX, including isoxazolidine derivatives, which are small molecule compounds that mimic the function of the TADs of transcriptional activators, and natural products such as sekikaic acid and lobaric acid^21,22^. However, these small compounds have a weak binding affinity for KIX due to their small surface area; the half-maximal effective concentration (EC_50_) for the inhibition of the KIX–MLL interaction is 36 μM for the isoxazolidine derivative^21^, while a half-maximal inhibitory concentration (IC_50_) is 34 μM and 17 μM for sekikaic acid and lobaric acid, respectively^22^. These values are approximately 10- to 20-fold larger than the dissociation constant (*K*_d_) for the binding between KIX and MLL TAD (*K*_d_ = 2.1 μM)^23^. Hence, developing a peptide inhibitor that can bind KIX more tightly and have a sufficient inhibitory effect on the KIX–MLL interaction is necessary.

MLL promotes transcription when both the TAD and DNA-binding domain of MLL simultaneously bind KIX and the target DNA, respectively. Thus, peptide inhibitors that are designed using only the MLL TAD fragment are expected to be effective in inhibiting the interaction between the MLL site of KIX and transcriptional activators without inducing transcription. Based on a similar idea, we have recently succeeded in developing a peptide that can inhibit the interaction between KIX and c-Myb by introducing amino-acid substitutions in the TAD fragment of c-Myb to enhance the binding affinity for the c-Myb site of KIX^24^. In this study, using the MLL TAD fragment as a template, we rationally designed peptide inhibitors that are predicted to bind the MLL site of KIX more tightly than the wild-type MLL by introducing amino-acid substitutions into the MLL TAD peptide using the Rosetta protein design software suite^25^. We demonstrated that one of the designed peptides, the T2857W mutant of the MLL TAD peptide, has a ~ twofold higher affinity for KIX than the wild-type MLL and is effective in inhibiting the KIX–MLL interaction.

## Results

### Computational design using Rosetta

First, the structure of the KIX:MLL:c-Myb ternary complex, which was determined by nuclear magnetic resonance (NMR) (PDB ID: 2AGH; containing 87 residues of KIX, 31 residues of MLL TAD, and 25 residues of c-Myb TAD)^15^, was energetically optimized using the PackRotamersMover (repack) and FastRelax (relax) modules of the Rosetta software suite^25^ (Supplementary Fig. 1). The repack calculations were used to select optimal side chains from rotamer libraries with the main chain fixed, and subsequently the relax calculations were used to minimize the total energy of the system by fine-tuning the complex structure initially with the main-chain restraint and then without it. These calculations were independently performed approximately 2000 times for each of the 20 model structures in the PDB file. The results indicated that among the 20 model structures, model 17 was the most stable, with the lowest “total_score”, which indicates the stability of the whole complex structure. From the ~ 2000 energetically optimized structures of model 17, the structure with the lowest “dG_separated” value, which indicates the binding energy between KIX and MLL, was selected as a template for subsequent amino-acid substitutions.

**Figure 2:**
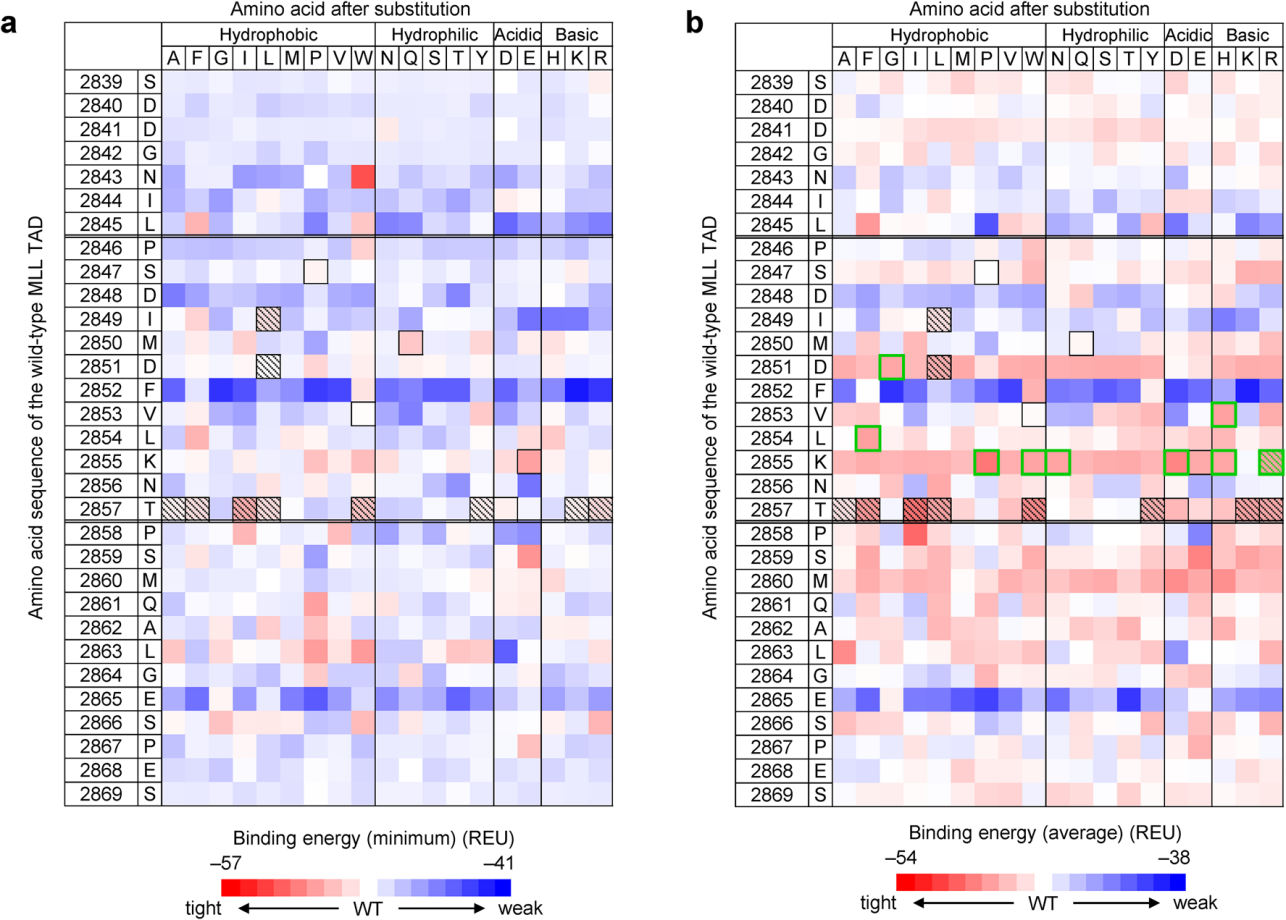
The KIX-binding energies of MLL TAD mutants predicted by saturation mutagenesis using Rosetta. Binding energy (in Rosetta energy unit [REU]) of a mutant represented by a dG_separated value is shown with color gradient from red (tighter binding than the wild type [WT]) to blue (weaker binding than the WT). Left columns show the residue number and the wild-type residue, and top rows show the substituted residue. For each mutant, Rosetta calculation (fixbb) was performed ~ 80 times, and the minimum (**a**) and average (**b**) of the dG_separated values for each mutant are shown. Residues 2846–2857 of the MLL TAD form a helical structure when bound to KIX. Black squares show the mutants selected based on the minimum dG_separated values. In (**b**), green squares show the mutants additionally selected based on the average dG_separated values. The mutants selected for experimental characterization are shown by hatched boxes. The figures were created with Microsoft Excel for Microsoft 365 (Microsoft Corp., Redmond, WA, USA; https://www.office.com).

Next, to search for single mutants of the MLL TAD peptide with only one amino acid substitution, we performed computational saturation mutagenesis, in which each of the 31 residues of the MLL TAD (residues 2839–2869) was replaced, one at a time, with one of 18 different amino acids other than cysteine and the wild-type residue (a total of 558 mutants [= 31 sites × 18 types]). Here, amino acid substitutions were introduced using the PackRotamersMover (fixbb) module of the Rosetta software suite, the total energy of the complex structure was minimized with the relax module, and then a dG_separated value was obtained. Such calculations were performed ~ 80 times for each mutant. The average and minimum of the dG_separated values for each mutant were compared with those of the wild type (Fig. 2). We found that the dG_separated values were little affected by the mutations at the residues in the N- and C-terminal regions of the MLL TAD but were significantly affected by those at the central α-helix (residues 2846–2857) and its neighboring residues, which interact directly with KIX (Fig. 2). In particular, the mutations around the C-terminal half of the α-helix were predicted to enhance the interaction between KIX and MLL. Note that the mutations at F2852 of MLL, which has hydrophobic interactions with F612, M625, and L628 of KIX, were not predicted to improve the KIX-binding affinity except for the substitution to tryptophan (Fig. 2). This suggests that F2852 of MLL is important for the binding to KIX, which is consistent with previous studies^23,26^.

To design the peptides that are expected to have higher KIX-binding affinity than the wild-type MLL, we selected the mutations with lower total_score and lower dG_separated values than the wild type (Supplementary Fig. 2). The mutation sites were restricted to the helical region of the MLL TAD that directly interacts with KIX, because the loop regions on both sides of the α-helix are flexible and unlikely to form stable interactions with KIX. Among the mutants, 29 had a minimum dG_separated value that was lower than the wild type, and 15 of the 29 had mutations in the helical region (Fig. 2 [black squares] and Supplementary Fig. 2b). These 15 mutants were considered to have an improved KIX-binding affinity. Among all mutants, 265 had an average dG_separated value that was lower than the wild type, and 114 of the 265 had mutations in the helical region (including the 15 mutants selected above) (Fig. 2b and Supplementary Fig. 2d). Among them, we further selected nine mutants with the lowest average dG_separated values (Fig. 2b [green squares], Supplementary Fig. 2d [red filled circles]). Thus, a total of 24 MLL TAD mutants were selected as candidates for the peptide inhibitors of the KIX–MLL interaction (Fig. 2 [black and green squares]).

### Mutant selection by helical propensity

**Figure 3:**
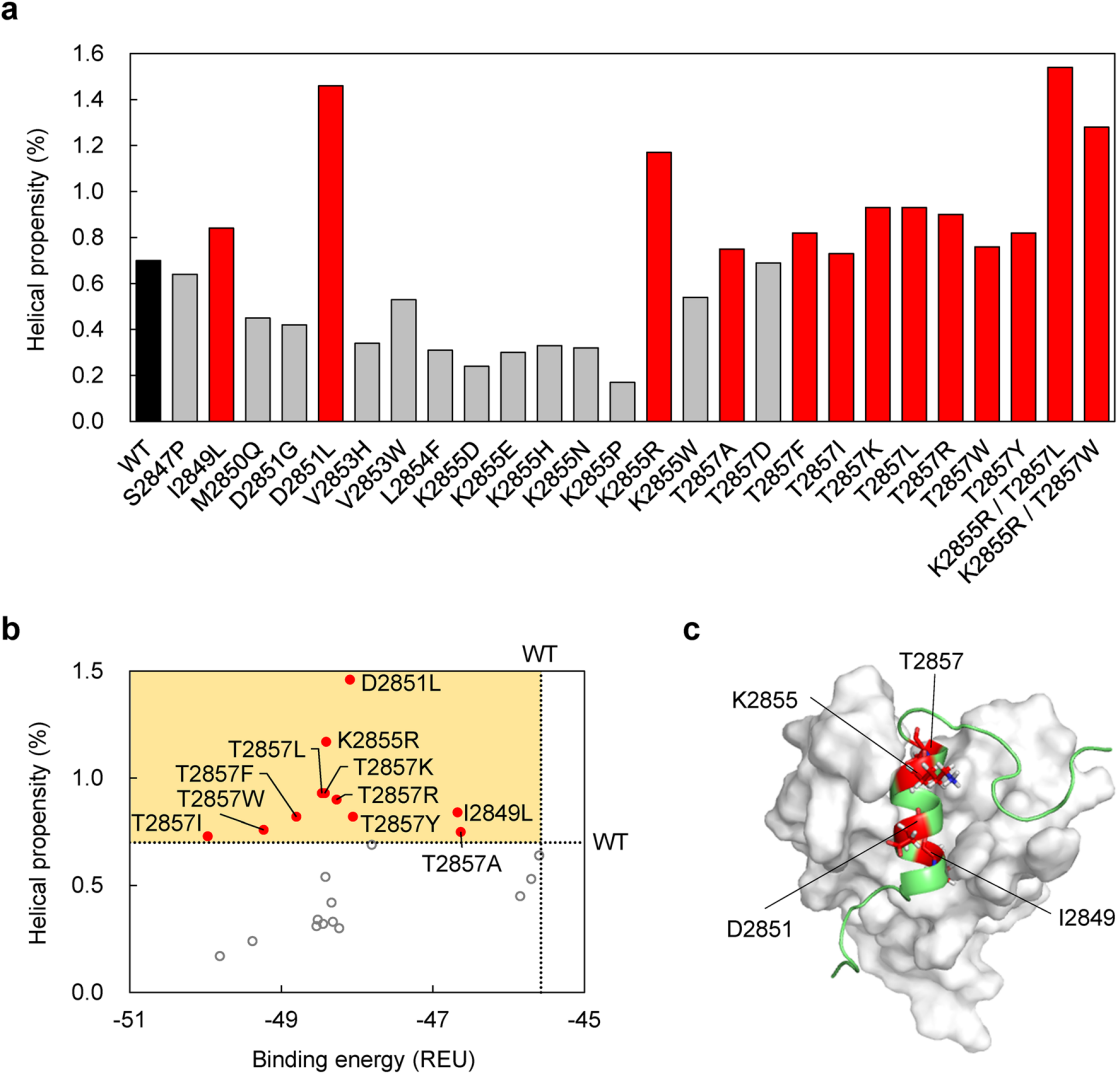
Selection of MLL TAD mutants. (**a**) The helical propensity of the wild type (WT, black) and mutants of MLL TAD predicted using AGADIR. The average of the residue-specific helical propensities (Supplementary Fig. 3) is shown. The mutants with higher or lower helical propensities than that of the WT are shown in red and grey, respectively. (**b**) The helical propensity plotted against the binding energy (average dG_separated value in Rosetta energy unit [REU]). The dotted lines show the values for the WT. Eleven single mutants (red filled circles) with a helical propensity higher than the wild type (in the yellow region) were selected for experimental characterization. (**c**) Four mutation sites for the 11 selected mutants are shown in red on the KIX:MLL complex structure (PDB ID: 2AGH). The figures were created with Microsoft Excel for Microsoft 365 (Microsoft Corp., Redmond, WA, USA; https://www.office.com) and with PyMOL Molecular Graphics System, Version 2.4.0 Schrödinger, LLC (https://pymol.org).

Previously, we successfully designed a peptide that tightly binds the c-Myb site of KIX by introducing mutations to stabilize the helical structure of the intrinsically disordered c-Myb TAD^24^. Similar to the c-Myb TAD, the MLL TAD is intrinsically disordered, and a decrease in the helical propensity may reduce the binding affinity for KIX. Thus, we evaluated the helical propensity of the aforementioned 24 mutants using the AGADIR server^27–30^ and selected the mutants with a helical propensity higher than that of the wild type. For the wild-type MLL TAD peptide, the helical propensity averaged over the entire peptide was ~ 0.7% (Fig. 3a), and the residue-specific helical propensity was the highest (~ 3%) at F2852 and V2853 (Supplementary Fig. 3). Eleven of the 24 mutants were predicted to have a higher helical propensity than the wild type (Fig. 3a,b). Therefore, these 11 mutants were selected for subsequent experimental characterization for KIX-binding affinity. Since eight of them had mutations at T2857, the mutations were introduced at four sites of the MLL TAD in this study (Fig. 3c).

### Secondary structure of designed peptides

**Figure 4:**
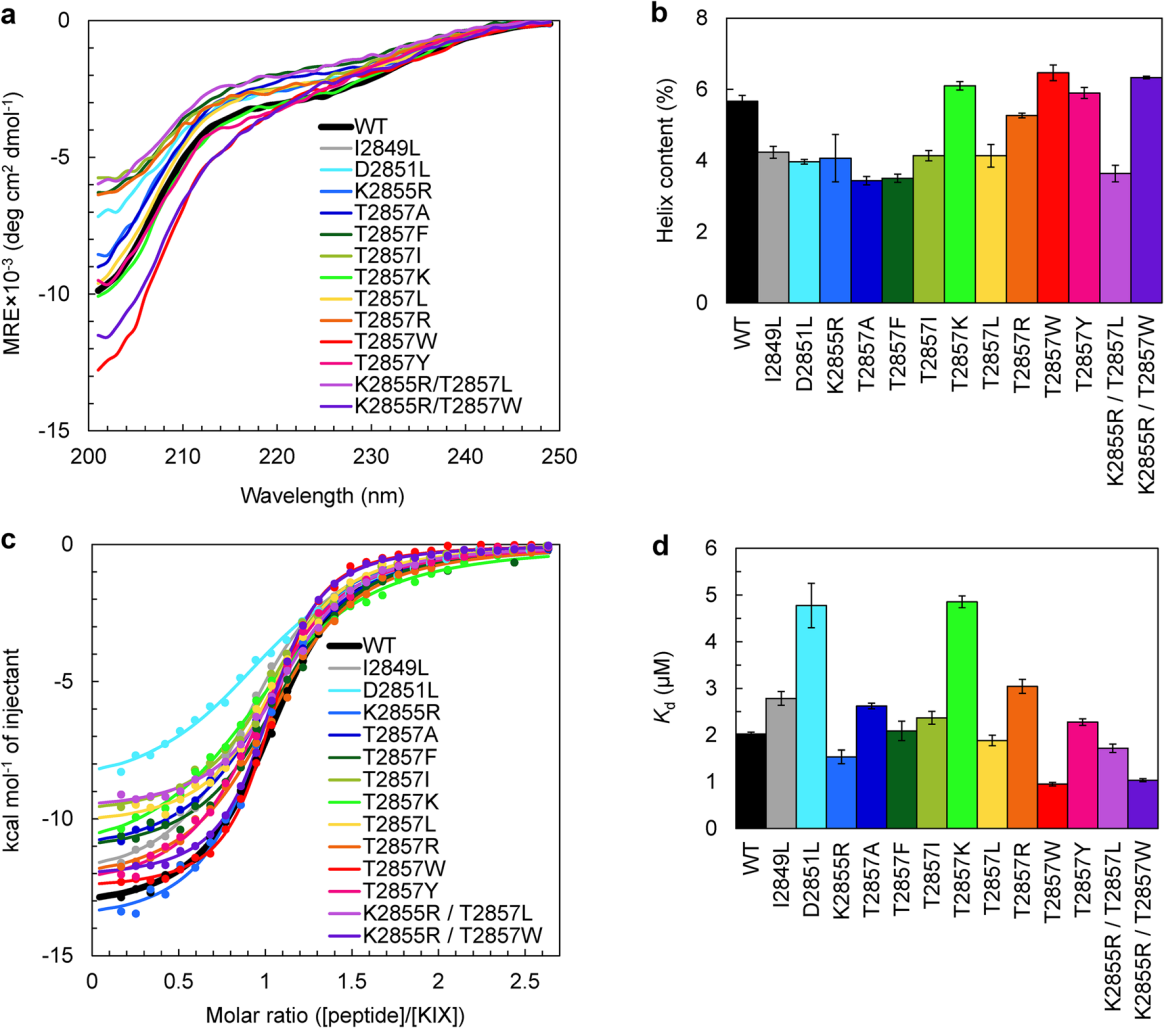
Secondary structure and KIX-binding affinity of the wild type and mutants of the MLL TAD. (**a**) Far-ultraviolet circular dichroism (CD) spectra of the MLL TAD peptides. (**b**) The helix contents estimated from the CD spectra. (**c**) KIX-binding isotherms of the wild type and mutants of the MLL TAD measured using isothermal titration calorimetry (ITC). The continuous lines are the curves fitted to a one-site binding model. (**d**) The *K*_d_ values obtained from the ITC measurements. In (**b**) and (**d**), the mean and standard error of triplicate or more measurements are shown. The figures were created with Microsoft Excel for Microsoft 365 (Microsoft Corp., Redmond, WA, USA; https://www.office.com).

The wild-type MLL TAD peptide as well as the 11 designed peptides (mutants of MLL TAD) were overexpressed in *Escherichia coli* as fusion proteins in which a 6 × His-tag and the B1 domain of *Streptococcal* protein G (GB1) were attached at the N-terminus of the MLL TAD. The His-tag–GB1 was removed from the TAD peptides via thrombin digestion during purification (see “Methods” section for details). The far-ultraviolet (UV) circular dichroism (CD) spectra of these peptides showed a decrease in the mean residue ellipticity (MRE) at low wavelengths, which is characteristic of disordered proteins (Fig. 4a). These spectra had a slight shoulder at 222 nm, but the intensity was small; the MRE at 222 nm was approximately −3000 deg cm^2^/dmol. The helix contents estimated from these values (see “Methods” section) were ~ 5.7% for the wild type and ~ 4–6% for the designed peptides, demonstrating that the designed peptides have helix contents comparable to that of the wild-type MLL TAD (Fig. 4b).

We also measured the CD spectrum of a mixture of the designed peptide and KIX and compared it with the sum of the CD spectra of the designed peptide alone and KIX alone (Supplementary Fig. 4). If two proteins did not interact, the CD spectrum of the mixture would coincide with the sum of the spectra of each protein. The measurements indicated that all CD spectra of the mixtures had more negative signals than the sum of the spectra of the isolated proteins, showing an increase in the helix contents. These results suggest that all 11 designed peptides interact with KIX to form a complex.

### KIX-binding affinity of designed peptides

The KIX-binding affinity of the designed peptides was determined using isothermal titration calorimetry (ITC) (Fig. 4c,d, Supplementary Figs. 5, 6, Table 1). The *K*_d_ value for the binding of the wild-type MLL TAD with KIX was 2.02 ± 0.04 μM, which is consistent with the previously reported value of 2.1 μM^23^. Of the 11 designed peptides, three peptides with either the K2855R, T2857L, or T2857W mutation exhibited a higher KIX-binding affinity than the wild type, with a *K*_d_ of 1.5 ± 0.1 μM, 1.9 ± 0.1 μM, and 0.95 ± 0.04 μM, respectively. In particular, the T2857W mutant had a ~ twofold higher affinity for KIX compared with the wild type. In contrast, the D2851L and T2857K mutants had a *K*_d_ of 4.8 ± 0.5 μM and 4.9 ± 0.1 μM, respectively, thus exhibiting ~ twofold lower affinity than the wild type (Fig. 4d).

**Table 1:** Thermodynamic parameters for KIX binding of the designed peptides.

MLL TAD peptide	*K*_d_ (μM)	Δ*H* (kcal/mol)	− *T*Δ*S* (kcal/mol)	Δ*G* (kcal/mol)	*N*
wild type	2.02 ± 0.04	− 13.1 ± 0.1	5.2 ± 0.1	− 7.91 ± 0.01	1.02 ± 0.01
I2849L	2.8 ± 0.1	− 12.3 ± 0.1	4.6 ± 0.2	− 7.70 ± 0.04	0.947 ± 0.008
D2851L	4.8 ± 0.5	− 8.8 ± 0.3	1.4 ± 0.4	− 7.39 ± 0.06	1.020 ± 0.006
K2855R	1.5 ± 0.1	− 13.8 ± 0.3	5.7 ± 0.4	− 8.07 ± 0.06	0.94 ± 0.02
T2857A	2.62 ± 0.06	− 11.8 ± 0.2	4.0 ± 0.2	− 7.74 ± 0.02	1.03 ± 0.02
T2857F	2.1 ± 0.2	− 11.1 ± 0.2	3.2 ± 0.3	− 7.88 ± 0.06	1.07 ± 0.01
T2857I	2.4 ± 0.1	− 10.02 ± 0.05	2.21 ± 0.08	− 7.80 ± 0.04	1.043 ± 0.003
T2857K	4.9 ± 0.1	− 11.7 ± 0.2	4.4 ± 0.2	− 7.37 ± 0.02	1.02 ± 0.02
T2857L	1.9 ± 0.1	− 10.51 ± 0.08	2.6 ± 0.1	− 7.94 ± 0.04	1.047 ± 0.009
T2857R	3.0 ± 0.2	− 12.6 ± 0.3	4.9 ± 0.3	− 7.66 ± 0.04	1.03 ± 0.01
T2857W	0.95 ± 0.04	− 12.78 ± 0.08	4.4 ± 0.1	− 8.35 ± 0.02	1.020 ± 0.006
T2857Y	2.28 ± 0.07	− 12.3 ± 0.2	4.5 ± 0.2	− 7.83 ± 0.02	1.02 ± 0.02
K2855R / T2857L	1.72 ± 0.09	− 9.5 ± 0.1	1.5 ± 0.2	− 8.00 ± 0.03	1.09 ± 0.01
K2855R / T2857W	1.04 ± 0.03	− 12.16 ± 0.06	3.86 ± 0.05	− 8.30 ± 0.02	1.04 ± 0.02

The dissociation constant (*K*_d_), changes in enthalpy (Δ*H*), entropy (Δ*S*), and Gibbs free energy (Δ*G*) upon binding, and stoichiometry of binding (*N*) were measured using isothermal titration calorimetry. The mean and standard error of triplicate or more measurements are shown. The *K*_d_ values are plotted in Fig. 4d, and other parameters are plotted in Supplementary Fig. 6.

Thermodynamic parameters indicated that the interaction between KIX and MLL is enthalpy driven (Supplementary Fig. 6, Table 1). The free energy change that occurred upon binding of KIX with a MLL TAD peptide (Δ*G* < 0) was the sum of the negative enthalpy change upon binding (Δ*H* < 0), which is favorable for binding, and the negative entropy change upon binding (Δ*S* < 0, i.e., − *T*Δ*S* > 0), which opposes binding, for both the wild-type and designed peptides (Supplementary Fig. 6, Table 1). The increase in the KIX-binding affinity by mutations was mainly attributed to the reduced contribution of the unfavorable entropy term for the T2857L and T2857W mutants and to the enhanced contribution of the favorable enthalpy term for the K2855R mutant. Thus, we obtained three peptides that bind KIX more tightly than the wild-type MLL TAD, especially the T2857W mutant of the MLL TAD peptide, which had a ~ twofold higher binding affinity than the wild type (Fig. 4d).

### Double mutants

We attempted to further enhance the KIX-binding affinity of the designed peptides by combining single mutations that enhanced KIX binding (K2855R, T2857L, and T2857W) to create double mutants (K2855R/T2857L and K2855R/T2857W). The Rosetta calculations indicated that the minimum dG_separated values for both the K2855R/T2857L and K2855R/T2857W double mutants are lower than that of the T2857W single mutant, suggesting the high KIX-binding affinities of the double mutants (Supplementary Fig. 7a). The AGADIR prediction indicated that the helical propensities of the double mutants are higher than those of the wild type and single mutants (Fig. 3a).

We found that the CD spectra and helix contents of the double mutants were similar to those of the single mutants and wild type (Fig. 4a,b). The CD spectrum of a mixture of the double mutant and KIX suggested the formation of a complex between them (Supplementary Fig. 4). The *K*_d_ values for KIX binding measured using ITC were 1.72 ± 0.09 µM and 1.04 ± 0.03 µM for the K2855R/T2857L and K2855R/T2857W mutants, respectively (Fig. 4c,d, Supplementary Fig. 5, Table 1). The *K*_d_ of the K2855R/T2857L mutant was an intermediate value between those of the K2855R (1.5 ± 0.1 µM) and T2857L mutants (1.9 ± 0.1 µM). Similarly, the *K*_d_ of the K2855R/T2857W mutant was an intermediate value between those of the K2855R (1.5 ± 0.1 µM) and T2857W mutants (0.95 ± 0.04 µM). Thus, both double mutants had a KIX-binding affinity that was higher than the wild-type MLL TAD, but lower than the T2857W mutant. Thermodynamic parameters indicated that the contributions of both the enthalpy term that favors binding and the entropy term that opposes binding were reduced in the double mutants when compared to the constituent single mutants, resulting in weaker KIX binding than the T2857W mutant (Supplementary Fig. 6, Table 1). In summary, among the peptides designed in this study, the MLL TAD peptide with the T2857W single mutation had the highest binding affinity for KIX.

### Competitive inhibition assay

**Figure 5:**
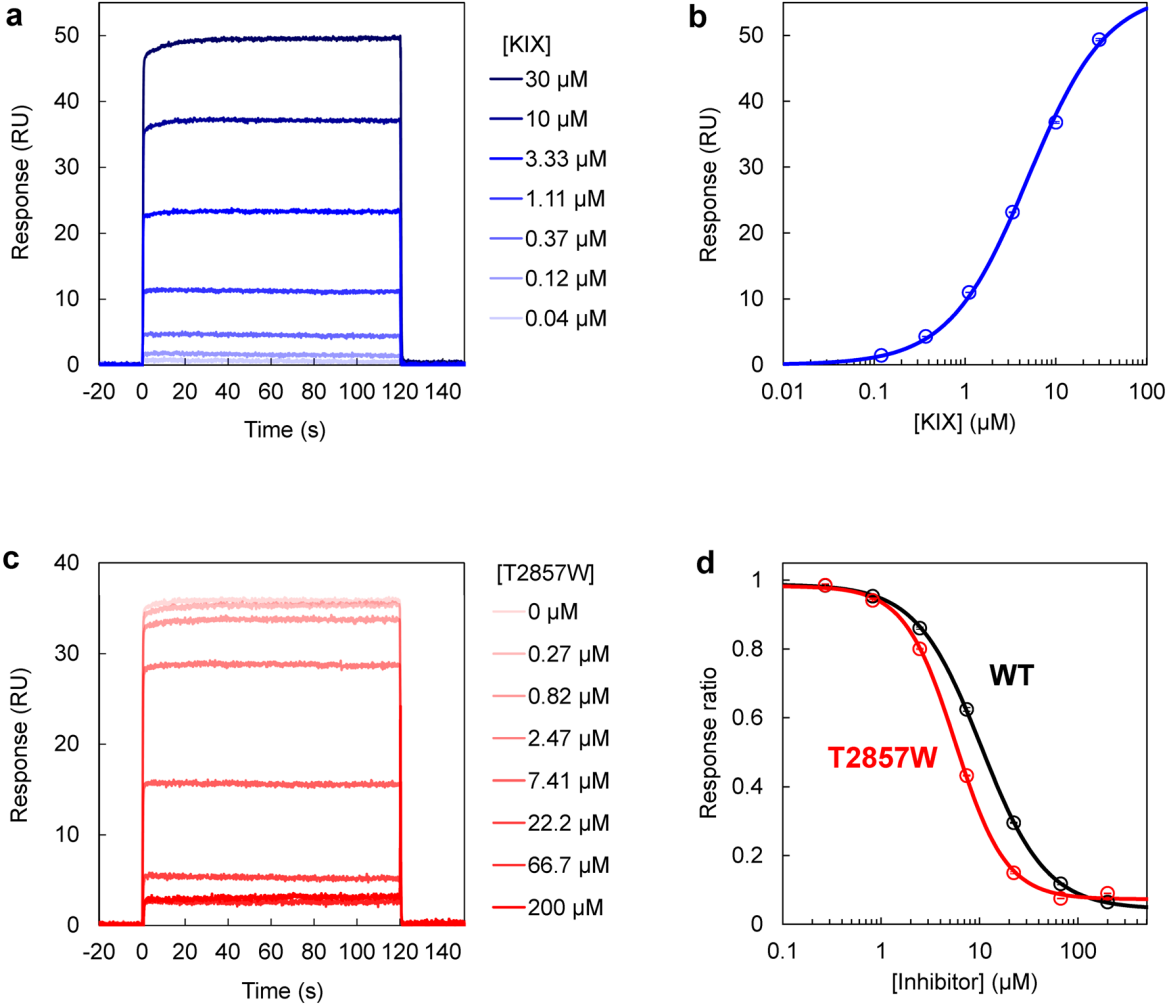
Competitive inhibition assay using surface plasmon resonance. (**a**) The sensorgrams of KIX binding to the immobilized MLL TAD. The KIX concentrations are shown. (**b**) The titration curve of the KIX binding to the immobilized MLL TAD. The continuous line was obtained by fitting to Eq. (4). (**c**) The sensorgrams of KIX binding to the immobilized MLL TAD in the presence of the designed T2857W peptide. The peptide concentrations are shown. The KIX concentration was 5 μM. (**d**) Inhibition of KIX binding to the immobilized MLL TAD by an increasing concentration of an inhibitor (the wild type [WT] and T2857W mutant of the MLL TAD peptide). The IC_50_ value was 10.4 ± 0.2 μM for WT and 5.67 ± 0.01 μM for the T2857W mutant. In (**b**) and (**d**), the mean and standard error of triplicate measurements are shown. The figures were created with Microsoft Excel for Microsoft 365 (Microsoft Corp., Redmond, WA, USA; https://www.office.com).

Then, we performed a competitive inhibition assay using surface plasmon resonance (SPR) to obtain an IC_50_ value for the designed T2857W peptide. First, to confirm the binding of KIX to MLL, which was immobilized on a sensor chip of SPR, we measured the interaction between KIX and the wild-type MLL TAD using SPR. The *K*_d_ value obtained was 4.94 ± 0.02 μM (Fig. 5a,b), which was ~ twofold larger than that measured using ITC (*K*_d_ = 2.02 ± 0.04 μM; Fig. 4c,d). Next, we injected a mixture of KIX and the designed peptide onto the immobilized MLL. KIX bound to the immobilized MLL when the concentration of the inhibitor (the designed peptide) was low. As the inhibitor concentration increased, the binding of KIX to the immobilized MLL was reduced (Fig. 5c). The IC_50_ value obtained from this assay was 5.67 ± 0.01 μM for the designed T2857W peptide (Fig. 5d). When the wild-type MLL TAD peptide was used as an inhibitor, the IC_50_ value was 10.4 ± 0.2 μM (Fig. 5d, Supplementary Fig. 8). Thus, the designed T2857W peptide has ~ twofold higher inhibitory effect when compared to the wild-type peptide.

**Figure 6:**
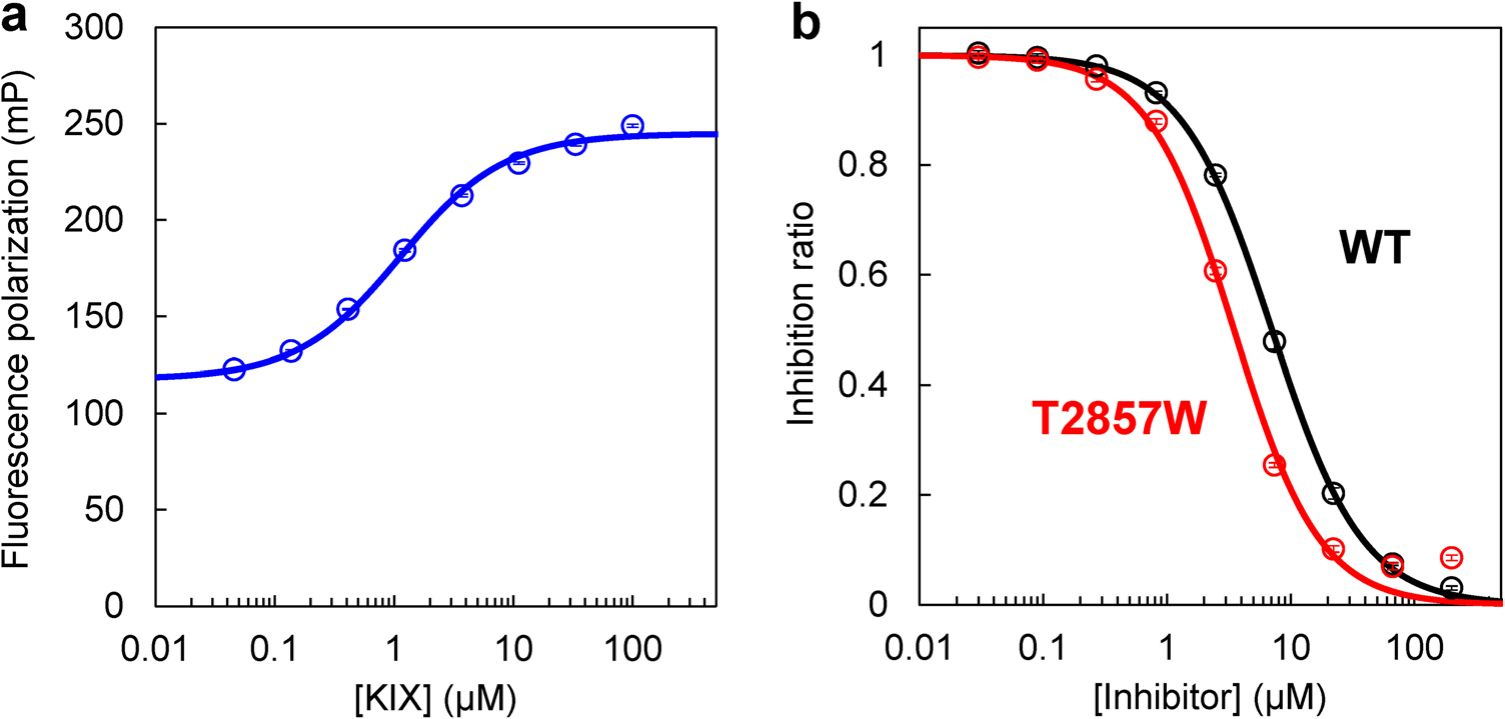
Competitive inhibition assay using fluorescence polarization. (**a**) The titration curve of the KIX binding to the FITC-labeled MLL TAD peptide (FITC-MLL). The continuous line was obtained by fitting to Eq. (6). (**b**) Inhibition of KIX binding to FITC-MLL by an increasing concentration of an inhibitor (the wild type [WT] and T2857W mutant of the MLL TAD peptide). The IC_50_ value was 7.1 ± 0.3 μM for WT and 3.48 ± 0.05 μM for the T2857W mutant. The mean and standard error of triplicate measurements are shown. The figures were created with Microsoft Excel for Microsoft 365 (Microsoft Corp., Redmond, WA, USA; https://www.office.com).

A competitive inhibition assay was also performed using fluorescence polarization. To confirm the binding of KIX to the fluorescein isothiocyanate (FITC)-labeled MLL TAD peptide (FITC-MLL), we titrated KIX into the FITC-MLL. The fluorescence polarization of FITC-MLL increased upon binding to KIX (Fig. 6a). The *K*_d_ for the interaction between KIX and FITC-MLL measured by fluorescence polarization was 1.13 ± 0.01 μM, which was slightly smaller than those measured by ITC (2.02 ± 0.04 μM; Fig. 4c,d) and SPR (4.94 ± 0.02 μM; Fig. 5a,b). This may be due to the attachment of the FITC label to the MLL peptide. Next, competitive inhibition experiment was performed by titrating the designed T2857W peptide into a mixture of KIX and FITC-MLL. As the inhibitor concentration increased, the fluorescence polarization was decreased (Fig. 6b), indicating the dissociation of KIX from FITC-MLL. The IC_50_ value thus obtained for the designed peptide was 3.48 ± 0.05 μM, showing ~ twofold higher inhibitory effect compared to the wild-type peptide (IC_50_ = 7.1 ± 0.3 μM; Fig. 6b). These results are consistent with those obtained using SPR.

### Binding specificity of the designed peptide to the MLL site of KIX

KIX has two binding sites for transcriptional activators: the MLL site and c-Myb site. To test whether the designed T2857W peptide also inhibits the c-Myb–KIX interaction, we performed a competitive inhibition assay using the FITC-labeled c-Myb TAD peptide (FITC-Myb). The binding of FITC-Myb to KIX was confirmed by titration experiments (Supplementary Fig. 9a). Then, we titrated the wild-type MLL TAD into a mixture of KIX and FITC-Myb. As the MLL concentration increased, the fluorescence polarization was decreased, indicating that the wild-type MLL can inhibit c-Myb binding to KIX (IC_50_ = 36 ± 1 μM; Supplementary Fig. 9b). This is not surprising since MLL is known to bind the c-Myb site of KIX, but with much lower affinity than c-Myb^23^. The IC_50_ of the designed T2857W peptide against the interaction between KIX and FITC-Myb was 16 ± 1 μM (Supplementary Fig. 9b). However, these IC_50_ values for c-Myb binding to KIX are ~ fivefold higher than those for MLL binding to KIX, suggesting that the designed inhibitor is more specific for the MLL–KIX interaction than the c-Myb–KIX interaction.

## Discussion

**Figure 7:**
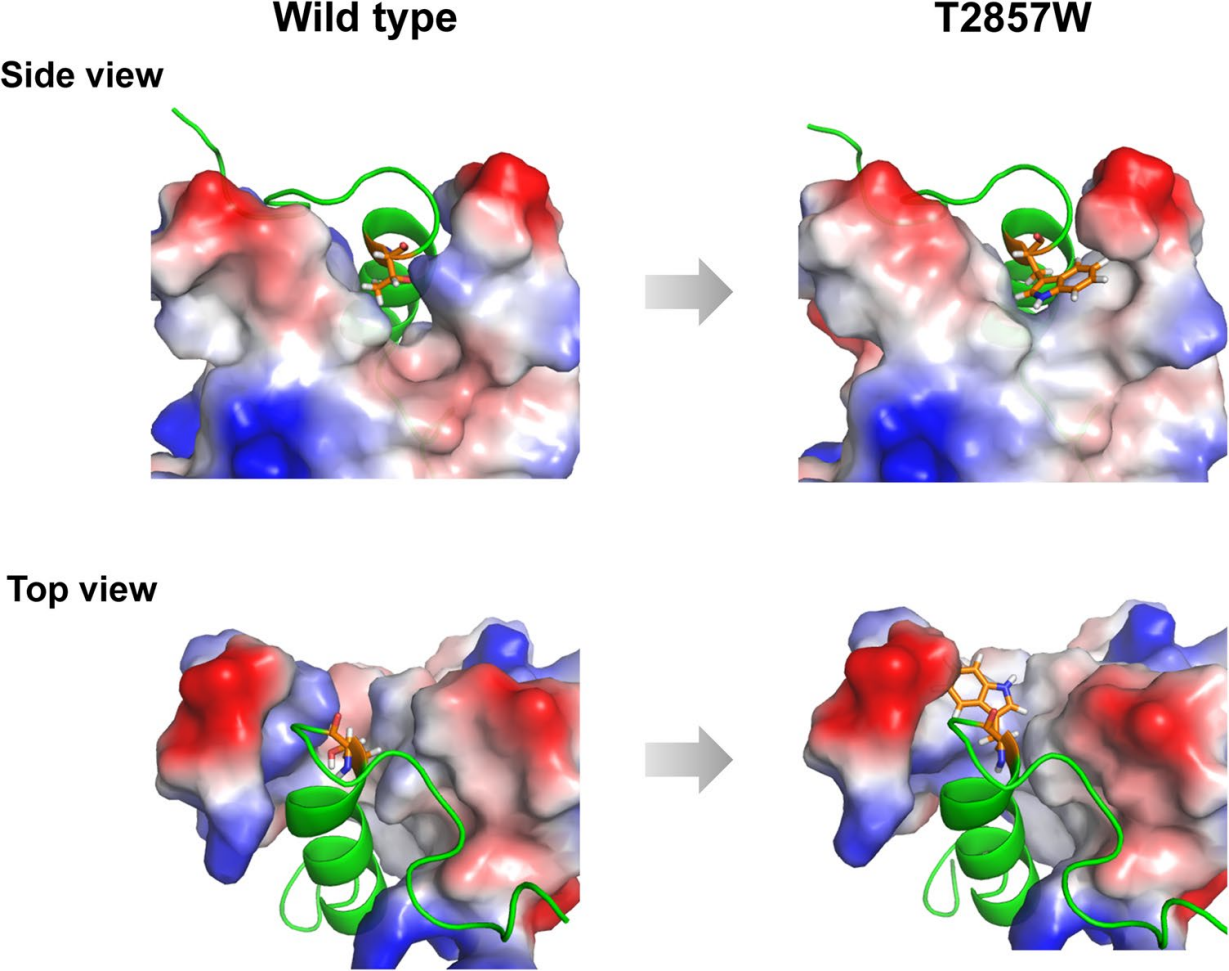
Comparison of the KIX:MLL complex structure for the wild type (left) and T2857W mutant of MLL (right). The complex structures were modeled using Rosetta. MLL is shown in green, and residue 2857 (Thr for the wild type and Trp for the mutant) is shown in orange. KIX is shown in gray, red, and blue for hydrophobic, negative, and positive surfaces, respectively. The figure was drawn using PyMOL Molecular Graphics System, Version 2.4.0 Schrödinger, LLC (https://pymol.org).

In this study, we rationally designed peptides that tightly bind the MLL site of KIX and inhibit the KIX–MLL interaction by introducing mutations into the MLL TAD fragment. We demonstrated that the T2857W mutant of the MLL TAD peptide was able to successfully inhibit the KIX–MLL interaction. The inhibitor has ~ twofold higher binding affinity for KIX and ~ twofold higher inhibitory effect on the KIX–MLL interaction than the wild-type MLL TAD peptide. Since the MLL site of KIX binds various transcriptional activators, the designed peptide may also inhibit the interactions between them; however, this remains to be clarified. The MLL TAD is known to bind a large hydrophobic groove of KIX^31^. The model structure of the T2857W mutant created by Rosetta suggests that the substituted Trp residue enhances the hydrophobic interactions between KIX and MLL by burying the hydrophobic surface of KIX, thereby increasing the KIX-binding affinity (Fig. 7). The ITC measurements confirmed this by demonstrating that the T2857W mutation is more entropically favorable for KIX binding than the wild type (Supplementary Fig. 6, Table 1). Since the formation of hydrophobic contacts are known to be driven by an increase in entropy due to the release of water molecules surrounding the hydrophobic residues, the present thermodynamic data suggest that the T2857W mutation enhances hydrophobic interactions.

Previous studies have reported small molecule inhibitors of the KIX–MLL interaction, including the isoxazolidine derivative (EC_50_ = 36 μM)^17^, sekikaic acid (IC_50_ = 34 μM)^22^, lobaric acid (IC_50_ = 17 μM)^22^, and KG-501 (IC_50_ = 200 ± 10 μM)^32^. However, the EC_50_ and IC_50_ values of these small molecule inhibitors are 10- to 100-fold larger than the *K*_d_ for the KIX–MLL interaction (2.02 ± 0.04 μM). In contrast, the IC_50_ of the T2857W peptide (5.67 ± 0.01 μM) obtained in this study is comparable to the *K*_d_ for the KIX–MLL interaction (2.02 ± 0.04 μM) and therefore is significantly better than those of the small molecules. Previous studies have also reported peptide-based inhibitors, including a peptide in which a disulfide crosslink was introduced into MLL TAD to improve the peptide stability^33^ and a peptide in which non-natural amino acids were introduced into the MLL TAD using the AlphaSpace software^31,34^; however, the KIX-binding affinity of these peptides was lower than that for the wild-type MLL TAD peptide. The *K*_d_ value obtained for the T2857W peptide in our study (0.95 ± 0.04 μM) is substantially better, thus facilitating tighter KIX binding. Therefore, to the best of our knowledge, the T2857W peptide developed in this study has the highest affinity for KIX among the previously reported small molecule- and peptide-based inhibitors that target only the MLL site of KIX.

Interestingly, Joy et al. recently developed a peptide-based inhibitor of PPIs at the c-Myb site of KIX, named MybLL-tide, in which the c-Myb TAD peptide and MLL TAD peptide are crosslinked by 8-amino-3,6-dioxaoctanoic acid^35^. Although the inhibitor is longer than our designed peptide, it binds very tightly to KIX with a *K*_d_ of 380 ± 50 pM^35^. It may be possible that an introduction of both the T2857W mutation (found in the present study) and three Lys-to-Arg mutations (found in our previous study for designing inhibitors of the c-Myb–KIX interaction^24^) in the MLL TAD and c-Myb TAD regions of MybLL-tide, respectively, may further improve its KIX-binding affinity. Thus, we believe that developing PPI inhibitors using peptides is an attractive approach to drug discovery, and the present study may be useful for the rational development of peptides that are able to inhibit the interactions of the KIX domain of CBP with various transcriptional activators.

Although the designed T2857W peptide has a high inhibitory effect on the KIX–MLL interaction, the KIX-binding affinity may be further improved using the following three approaches. The first approach is the introduction of mutations in the N-terminal flexible region of MLL TAD, in addition to the helical region, as the deletion of the N-terminal region from the MLL TAD peptide is reported to reduce its binding affinity for KIX^31^. The second approach is the introduction of multiple mutations into the designed peptide. In the present study, the double mutants did not exhibit an improved KIX-binding affinity when compared to the single mutants, possibly because the mutation sites are only two residues apart, thus allowing interactions between the mutations, which may affect the KIX-binding affinity. Previous studies demonstrated that simultaneous mutations at distant sites in a protein can additively improve its function^36^. Thus, introducing both T2857W and distant mutation(s), especially in the N-terminal region of the designed peptide, may additively improve the KIX-binding affinity. The third approach is the introduction of mutations that increase the helical propensity of the designed peptide. We have recently succeeded in designing a helical peptide with enhanced binding to the c-Myb site of KIX by introducing Lys-to-Arg conservative substitutions on the helix surface opposite to the KIX binding interface to increase the helical propensity of the c-Myb TAD peptide^24^. In the present study, although the helix contents of the wild type and mutants of the MLL TAD peptide did not correlate with the Δ*G* upon KIX binding (Supplementary Fig. 7b), it may be possible to further improve the KIX-binding affinity of the designed peptide by introducing mutations on the helix surface opposite to the KIX-binding interface to enhance the helical propensity. Such a stabilization of the helical peptide may also improve short half-lives of peptide-based drugs. Further improvements of our peptide inhibitor using these strategies will be a future challenge. In addition, since the KIX–MLL interaction occurs inside cells, the fusion of a cell-penetrating peptide to the designed peptide may be necessary to facilitate translocation into cells^37^.

The Rosetta software is widely used for protein design; however, the correlation between Rosetta scores and experimental results is not high. In the present study, the correlation between the dG_separated values obtained by Rosetta and the Δ*G* obtained via ITC experiments was low (correlation coefficient *r* = 0.42–0.47; Supplementary Fig. 7a,c). Nevertheless, the T2857W mutant, which exhibited the highest KIX-binding affinity, had the third-best dG_separated (minimum) values among the single mutations within the helical region of the MLL TAD (Supplementary Fig. 2b). Therefore, although experimental results may not strictly correlate with Rosetta scores, it is suggested that a pool of mutations predicted to enhance target binding using Rosetta includes mutants that will be experimentally shown to have an enhanced binding affinity. Thus, the use of the Rosetta software suite, in combination with the AGADIR server for helical propensity prediction, would allow for more efficient screening of binding proteins when compared to a conventional directed evolution method that requires exhaustive experiments.

In conclusion, we succeeded in developing a helical peptide that inhibits the KIX–MLL interaction using a combination of theoretical saturation mutagenesis by Rosetta and helical propensity prediction by AGADIR. Thus, our approach may be useful for the rational development of helical peptide inhibitors of protein–protein interactions implicated in various diseases.

## Methods

### Rational design

Rational design of the MLL TAD mutants was performed with the Rosetta protein design software suite (version 3.8) using RosettaScripts^25,38,39^. The score function of talaris2014 was used^40^. The structures of the KIX:MLL:c-Myb ternary complex (PDB ID: 2AGH), determined by NMR, were used as input structures. For each of the 20 structures in the PDB file, side-chain optimization was performed using the PackRotamersMover (repack) module, and subsequent energy minimization was performed using the FastRelax (relax) module, initially with a backbone constraint and then without it. The “dG_separated” score, which indicates a binding energy between two proteins, was calculated using the InterfaceAnalyzerMover module^41^. A single amino acid substitution was introduced in MLL using the PackRotamersMover (fixbb) module, and subsequently the total energy of the complex structure was minimized with the relax module.

The helical propensities of MLL TAD peptides were predicted from amino-acid sequences using the AGADIR server^27–30^ (http://agadir.crg.es). The pH, temperature, and ionic strength were set to 7.0, 303 K, and 0.05 M, respectively, which were adjusted according to the experimental conditions.

### Protein expression and purification

The MLL TAD peptides and KIX protein were expressed and purified as described previously^24^. Briefly, the DNA fragment of MLL TAD (residues 2842–2869 of human MLL; the amino-acid sequence is GNILPSDIMDFVLKNTPSMQALGESPES), along with a 6 × His-tag (MGHHHHHHSSG), GB1, and thrombin cleavage site (LVPRG) fused at the N-terminus, was inserted into the *Nco*I and *Bam*HI restriction sites of the pET-15b expression vector (Merck Millipore, Darmstadt, Germany). A Tyr residue was attached at the C-terminus of the MLL TAD for protein concentration determination via UV absorption. The DNA fragment of KIX (residues 586–672 of mouse CBP), along with a 6 × His-tag and thrombin cleavage site at the N-terminus, was inserted into a pET-15b vector. The amino-acid sequence of mouse KIX is the same as that of human KIX. The genes of the MLL TAD mutants were constructed using the protocol of the QuikChange site-directed mutagenesis kit (Agilent Technologies, Santa Clara, CA, USA). The DNA sequences were confirmed by Eurofins Genomics (Tokyo, Japan).

For the expression of MLL TAD, *E. coli* BL21(DE3) competent cells were transformed with the plasmid and cultivated at 37 °C in 2 × YT liquid medium supplemented with ampicillin. When an optical density at 600 nm (OD_600_) of the cell culture reached 0.7–0.8, 1 mM isopropyl β-D-1-thiogalactopyranoside was added to induce protein expression. After 4 h of cultivation, the cells were harvested by centrifugation. The *E. coli* cell pellet was suspended with buffer HP (50 mM sodium phosphate [pH 7.4], 300 mM NaCl, 10 mM imidazole, and 6 M guanidine hydrochloride). The sonicated lysate was centrifuged at 4 °C, and the supernatant was filtered using a membrane filter and applied to a gravity column (50 mL) containing 5 mL of nickel-nitrilotriacetic acid (Ni–NTA) resin (Qiagen, Hilden, Germany). The column was washed with buffer HP and buffer AP (50 mM sodium phosphate [pH 7.4], 300 mM NaCl, and 10 mM imidazole) to remove impurities. To elute His-tag–GB1–MLL TAD for ITC and SPR measurements, 40 mL of buffer EP (50 mM sodium phosphate [pH 7.4], 300 mM NaCl, and 250 mM imidazole) was added to the column. In the ITC measurements, the wild-type MLL TAD peptides with and without His-tag–GB1 showed the same affinity for KIX. Thus, His-tag–GB1–MLL TAD was used in the ITC measurements, with the exception of the four mutants (I2849L, D2851L, K2855R, and T2857I). To obtain the MLL TAD peptide without His-tag–GB1 for CD measurements, we washed the column, to which the His-tag–GB1–MLL TAD was attached, with buffer TP (50 mM sodium phosphate [pH 7.4] and 300 mM NaCl), added 150 units of thrombin to the column for digestion of the His-tag–GB1, and eluted the MLL TAD peptide with buffer TP. After Ni–NTA column purification, anion exchange chromatography was performed to further purify the aforementioned four mutants without His-tag–GB1 using the AKTAprime plus chromatography system (Cytiva, Marlborough, MA, USA; 10 mM sodium phosphate [pH 6.0] and 0–1 M NaCl). Due to the fact that the CD spectra of the four mutants were unchanged before and after the anion exchange chromatography, we omitted this procedure for other mutants.

Expression and purification of the KIX protein was performed in the same way as MLL, except that *E. coli* BL21(DE3)pLysS competent cells and 2 × YT liquid medium with ampicillin and chloramphenicol were used. After Ni–NTA column purification, gel filtration chromatography was performed using a Superdex 200 pg column (Cytiva) and the ITC buffer (20 mM Tris–acetate [pH 7.0] and 50 mM NaCl).

The purity of the proteins was confirmed using sodium dodecyl sulfate–polyacrylamide gel electrophoresis. All proteins were concentrated using an Amicon^®^ Ultra-4 centrifugal filter (molecular weight cut-off of 3000 Da; Merck Millipore) and dialyzed with the ITC buffer before measurements.

### CD measurements

CD spectra were measured using a J-805 spectropolarimeter (JASCO, Tokyo, Japan) from 200 to 250 nm in a quartz cuvette with a 1-mm path length at 30 °C. Protein concentrations were 30 μM. The CD intensity was converted to MRE using the following equation:

1 $$MRE{\text{ (deg cm}}^{{2}} {\text{ dmol}}^{ - 1} {)} = \frac{{CD{\text{ (mdeg)}}}}{{N \cdot c{\text{ (M)}} \cdot l{\text{ (mm)}}}}$$

where *N* is the number of residues, *c* is the protein concentration, and *l* is the path length. For each peptide, the CD spectrum was measured in triplicate or more, and the averaged spectrum is shown in Fig. 4a.

The helix contents of the MLL TAD peptide were calculated from the far-UV CD spectra using the CONTINLL program^42^ with the reference data set containing soluble and denatured proteins (SDP48) in the CDPro package^43^.

### ITC measurements

ITC measurements were performed using MicroCal iTC200 (Malvern Panalytical Ltd., Malvern, United Kingdom) at 30 °C. Samples were sufficiently dialyzed with the ITC buffer before measurements. The 600 μM MLL solution was titrated into the cell containing 45 µM KIX. The data were analyzed using the Origin 7 software (OriginLab Corp., Northampton, MA, USA; https://www.originlab.com) to obtain the *K*_d_ (M), Δ*H* (kcal/mol), and stoichiometry of binding, *N*. The Δ*G* (kcal/mol) and Δ*S* (kcal/mol/K) were obtained as follows:

2 $$\Delta G = - RT\ln \left( {\frac{1}{{K_{{\text{d}}} }}} \right)$$

3 $$\Delta S \, = \frac{\Delta H - \Delta G \, }{T}$$

where *R* is the gas constant, and *T* is the temperature.

### SPR measurements

SPR measurements were performed using Biacore™ T100 (Cytiva) at 30 °C. All experiments were performed using the SPR buffer (20 mM sodium phosphate [pH 7.0], 100 mM NaCl, and 0.05% Tween 20). Immobilization of His-tag–GB1–MLL TAD to the Biacore Series S Sensor Chip CM5 (Cytiva) was conducted via amine coupling. A mixture of *N*-hydroxysuccinimide (NHS) and 1-ethyl-3-(3-dimethylaminopropyl)carbodiimide hydrochloride (Cytiva) was injected to activate the CM5 sensor chip, and subsequently the His-tag–GB1–MLL TAD diluted to 100 μg/mL in the immobilization buffer (10 mM sodium acetate [pH 5.5]; Cytiva) was injected and immobilized (77.9 RU). Ethanolamine (Cytiva) was injected to deactivate the remaining active NHS groups on the sensor chip. Then, KIX (0.04–30 μM) was injected to measure its binding to MLL, while a mixture of KIX (5 μM) and an inhibitor (0.27–200 μM) was injected to measure the IC_50_ of the inhibitor (contact time 120 s, flow rate 30 μL/min, and dissociation time 120 s). Each injection was followed by a wash with 10 mM HCl for the regeneration of the sensor chip (contact time 30 s and flow rate 30 μL/min).

All sensorgrams were analyzed using the Biacore T100 Evaluation Software (Cytiva). The response values at the end of each sample injection were used for equilibrium value analysis. *K*_d_ was obtained by fitting the titration data with the following equation:

4 $$R = \frac{{[{\text{KIX}}]}}{{[{\text{KIX}}] + K_{{\text{d}}} }}R_{\max }$$

where *R* is the observed response value, [KIX] is the concentration of KIX, and *R*_max_ is the maximum response. The IC_50_ values were obtained using the following equation:

5 $$R = R_{\min } + \frac{{R_{\max } - R_{\min } }}{{1 + \left( {\frac{{[{\text{Inhibitor}}]}}{{{\text{IC}}_{50} }}} \right)^{H} }}$$

where *R*_min_ is the minimum response, and *H* is the Hill coefficient^24^. The data fitting was performed using the KaleidaGraph 4.1 software (Synergy Software, Reading, PA, USA; https://www.synergy.com). The *K*_d_ and IC_50_ values were obtained via triplicate measurements, and the mean and standard error were determined.

### Fluorescence polarization measurements

Fluorescence polarization measurements were performed using an ARVO X5 multilabel plate reader (PerkinElmer, Waltham, MA, USA) at 30 °C with 96-well non-binding surface (NBS) polystyrene microplates (Corning Inc., Corning, NY, USA). All experiments were performed using the buffer containing 20 mM sodium phosphate (pH 7.0), 100 mM NaCl, and 0.005% Tween 20. For all measurements, 25 nM of FITC-labeled peptides were used. For KIX binding experiments, 0–100 μM KIX was titrated into the FITC-labeled peptide. For competitive inhibition experiments, an inhibitor (0–200 μM of the wild-type MLL TAD or designed T2857W peptide) was titrated into a mixture of KIX (2 μM) and the FITC-labeled peptide.

The FITC-labeled MLL and c-Myb TAD peptides with the following amino acid sequences (29 and 33 residues, respectively) were purchased from Biologica Co. (Aichi, Japan):

$${\text{FITC-MLL : }}\left( {{\text{FITC}}} \right){\text{-}}\left( {\beta {\text{-Alanine}}} \right){\text{-GNILPSDIMDFVLKNTPSMQALGESPESY}}$$

$${\text{FITC-Myb : }}\left( {{\text{FITC}}} \right){\text{-}}\left( {\beta {\text{-Alanine}}}\right){\text{-GYNDEDPEKEKRIKELELLLMSTENELKGQQAL}}$$

The *K*_d_ values for KIX binding of the FITC-labeled peptides were obtained by fitting the titration curves with the following equation:

6 $$r = r_{0} + \frac{\Delta r}{{2[{\text{A}}]}}\left( {[{\text{A}}] + [{\text{B}}] + K_{{\text{d}}} - \sqrt {([{\text{A}}] + [{\text{B}}] + K_{{\text{d}}} )^{2} - 4[{\text{A}}][{\text{B}}]} } \right)$$

where* r* is the observed fluorescence polarization of the FITC label, *r*_0_ is the fluorescence polarization in the absence of KIX, Δ*r* is the maximum change in fluorescence polarization, [A] is the concentration of the FITC-peptide (25 nM), and [B] is the concentration of KIX. The IC_50_ values were estimated as described above for the competitive inhibition experiments using SPR. The data fitting was performed using the KaleidaGraph 4.1 software (Synergy Software, Reading, PA, USA; https://www.synergy.com). The *K*_d_ and IC_50_ values were measured in triplicate, and the mean and standard error were determined.

## Supplementary Information

Supplementary Figures.

## Data Availability

The authors declare that all data supporting the findings of this study are available within the paper and its supplementary information file.
