# Supplementary material for: Rational peptide design for inhibition of the KIX–MLL interaction
Source: Sci Rep. 2023 Apr 18;13:6330. doi: 10.1038/s41598-023-32848-2 (PMC10113271; doi:10.1038/s41598-023-32848-2)
Supplement: Supplementary file 1 — Supplementary Figures. [file 41598_2023_32848_MOESM1_ESM.pdf]

Supplementary Information

Rational peptide design for inhibition of the KIX–MLL interaction

Nao Sato[1], Shunji Suetaka[1], Yuuki Hayashi[1,2], and Munehito Arai[1,3,*]

[1]Department of Life Sciences, Graduate School of Arts and Sciences, The University of Tokyo, 3-8-1 Komaba, Meguro, Tokyo 153-8902, Japan
[2]Environmental Science Center, The University of Tokyo, 7-3-1 Hongo, Bunkyo, Tokyo 113-0033, Japan
[3]Department of Physics, Graduate School of Science, The University of Tokyo, 3-8-1 Komaba, Meguro, Tokyo 153-8902, Japan

*Corresponding author: Munehito Arai, Ph.D.
Department of Life Sciences, Graduate School of Arts and Sciences, The University of Tokyo
3-8-1 Komaba, Meguro, Tokyo 153-8902, Japan
Phone: +81-3-5454-6751
E-mail: arai@bio.c.u-tokyo.ac.jp

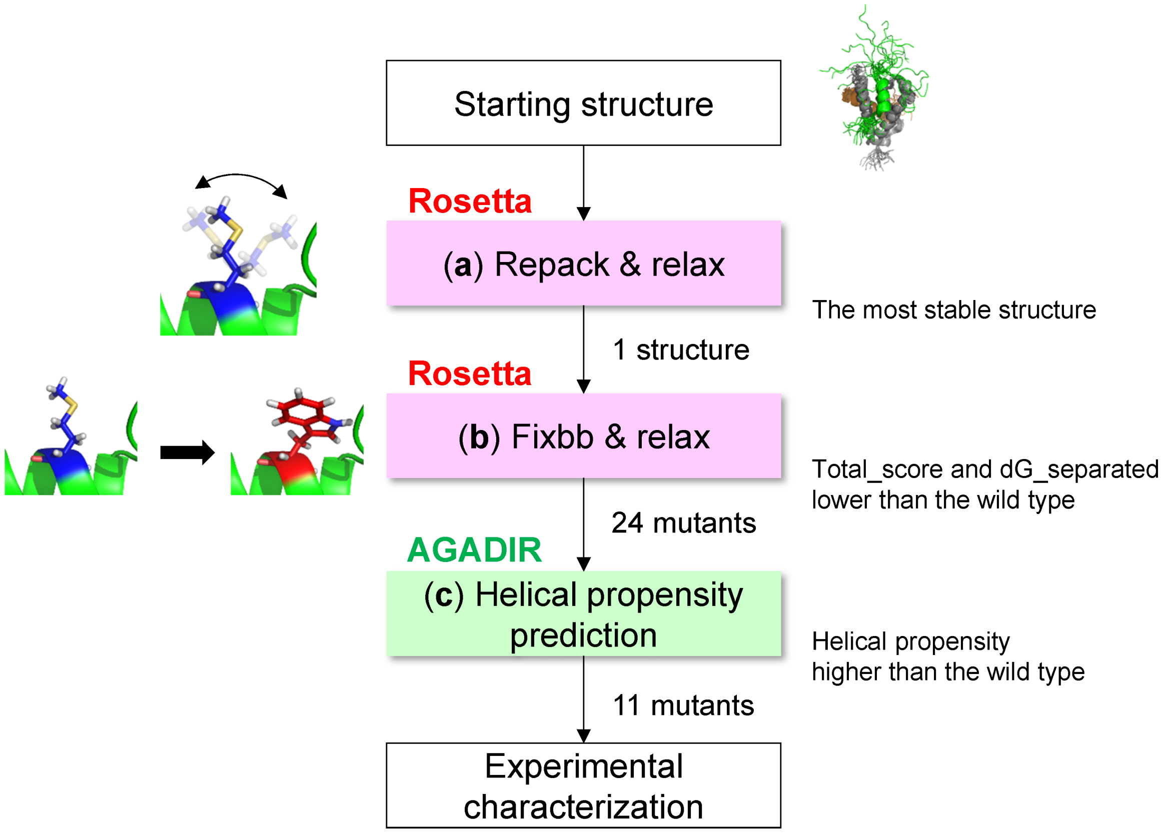

Supplementary Figure 1. Design workflow. (**a**) The structures of the KIX:MLL:c-Myb ternary complex (PDB ID: 2AGH) were energetically optimized using the repack and relax modules of the Rosetta software suite. The most stable structure with the lowest total_score value (model 17) was selected from 20 model structures in the PDB file. Then, from ~2000 energetically optimized structures of model 17, the structure with the lowest dG_separated value was selected. (**b**) Saturation mutagenesis for the MLL TAD was performed by introducing all types of amino acids (except Cys and the wild-type amino acid) at all positions one at a time using the fixbb and relax modules of Rosetta. Among the 558 mutants (31 sites × 18 types) of the MLL TAD, 24 mutants with lower total_score and dG_separated values than the wild type were selected. (**c**) The helical propensity was predicted by the AGADIR server. Among the 24 mutants, 11 mutants with helical propensities higher than that of the wild type were selected for experimental characterization. The figures were created with Microsoft PowerPoint for Microsoft 365 (Microsoft Corp., Redmond, WA, USA; https://www.office.com) and with PyMOL Molecular Graphics System, Version 2.4.0 Schrödinger, LLC (https://pymol.org).

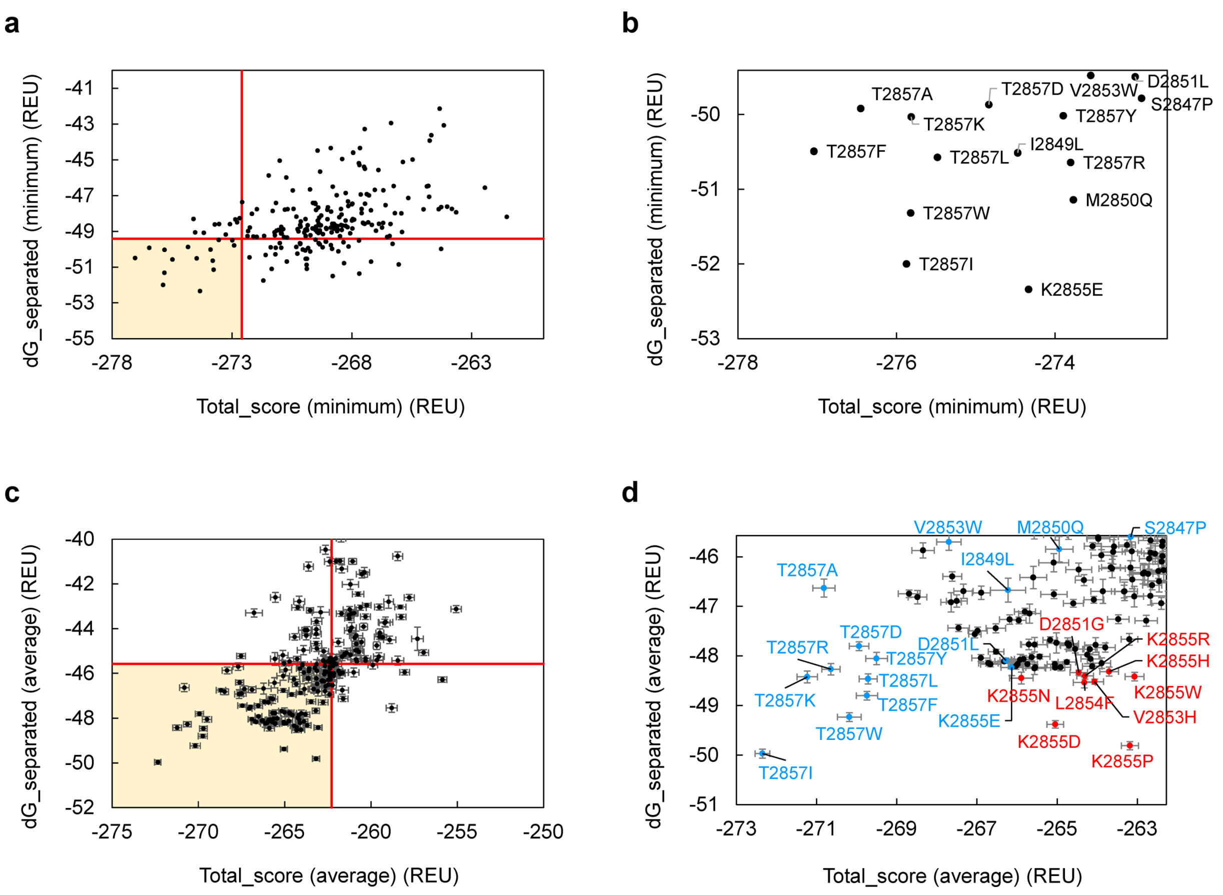

Supplementary Figure 2. Total_score and dG_separated values calculated by Rosetta for the mutations in the helical region of the MLL TAD (residues 2846–2857). (**a, b**) The minimum values of the total_score and dG_separated for each mutant among ~80-times Rosetta calculations (fixbb). The red lines show the values of the wild-type (WT) MLL TAD. The mutants in the bottom-left region (yellow) in (**a**) have lower total_score and dG_separated values than the WT. Close-up view of this region is shown in (**b**). (**c, d**) The average and standard error of total_score and dG_separated values of ~80-times Rosetta calculations (fixbb) for each mutant. The red lines show the values of the WT. The mutants in the bottom-left region (yellow) in (**c**) have lower total_score and dG_separated values than the WT. Close-up view of this region is shown in (**d**). In (**d**), the blue filled circles indicate the mutants selected based on the minimum dG_separated values, while the red filled circles indicate those additionally selected based on the average dG_separated values. The figures were created with Microsoft Excel for Microsoft 365 (Microsoft Corp., Redmond, WA, USA; https://www.office.com).

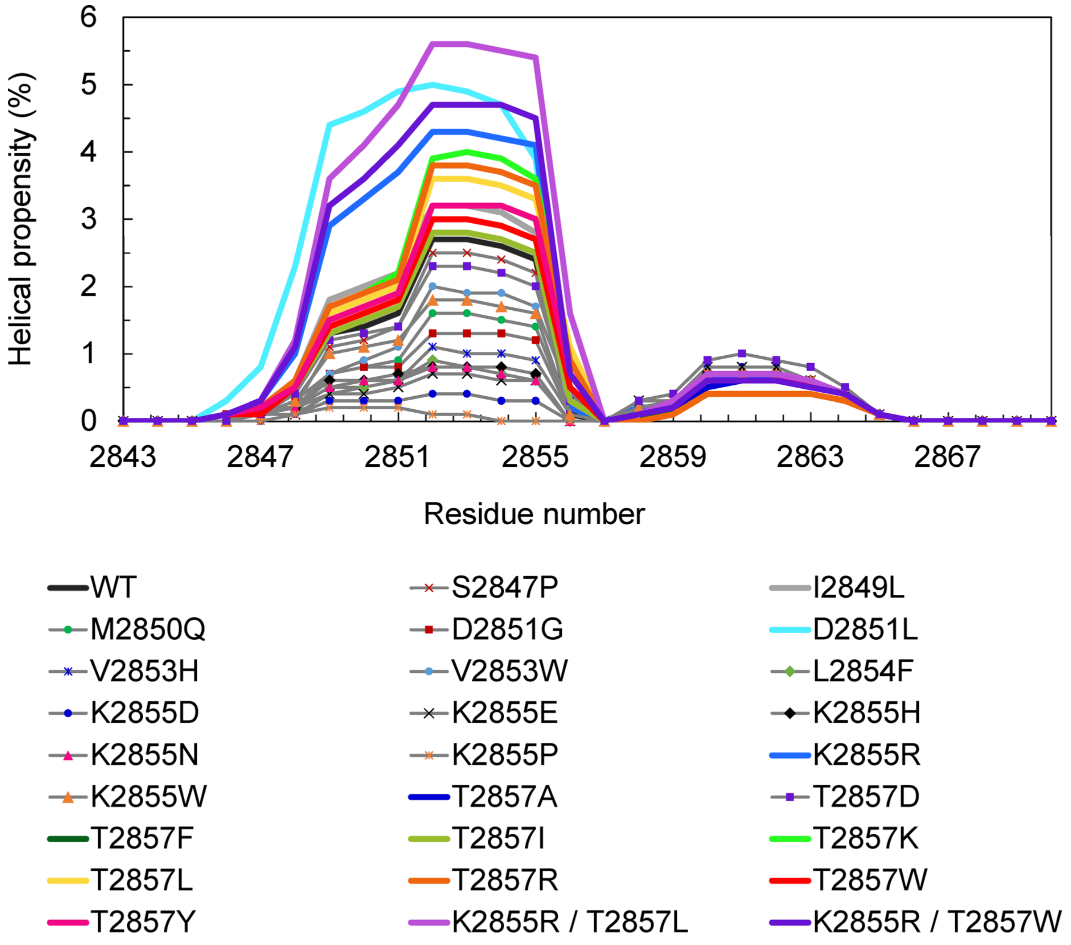

Supplementary Figure 3. Residue-specific helical propensity of the wild type and mutants of the MLL TAD peptide. The helical propensity was predicted using the AGADIR server. The residues that form an α-helix when bound to KIX (residues 2846–2857) demonstrate the high helical propensities. The 13 mutants and the wild type selected for experimental characterization are shown by thick lines, and other mutants are shown by thin lines with symbols. The figure was created with Microsoft Excel for Microsoft 365 (Microsoft Corp., Redmond, WA, USA; https://www.office.com).

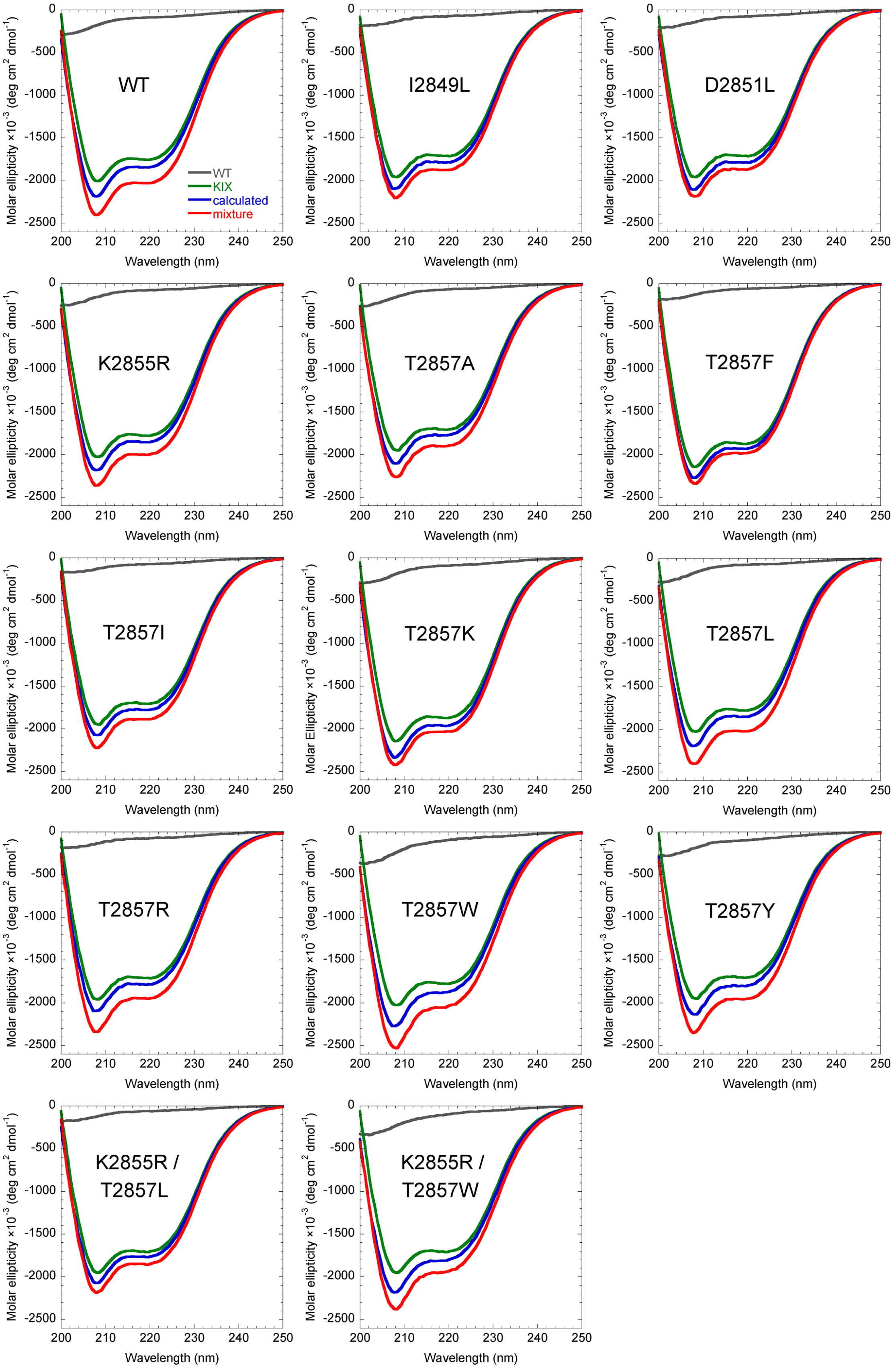

Supplementary Figure 4. Circular dichroism (CD) spectra of the MLL TAD peptide, KIX, and their mixture. The CD spectra of the MLL TAD peptide (gray), KIX (green), and a mixture of KIX and the MLL TAD peptide (red) are shown. The sum of the CD spectra of KIX alone and the MLL TAD peptide alone is shown in blue. The figures were created with KaleidaGraph 4.1.0 (Synergy Software, Reading, PA, USA; https://www.synergy.com).

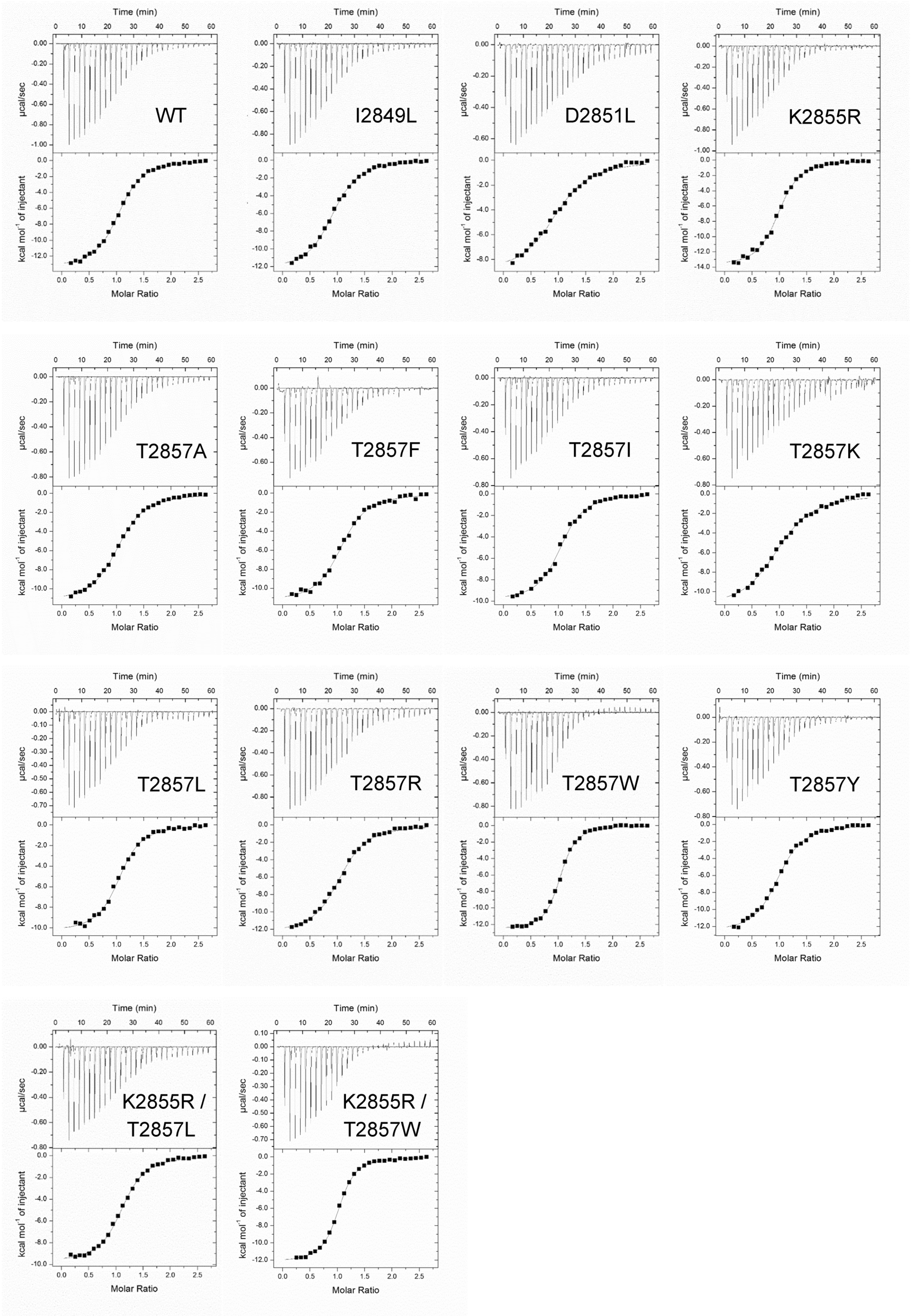

Supplementary Figure 5. Isothermal titration calorimetry measurements of KIX binding of the wild type and mutants of the MLL TAD. The upper panel shows the raw data of titrations, and the lower panel shows the integrated value of each titration peak plotted against the molar ratio ([MLL]/[KIX]). The continuous lines are obtained by fitting the binding isotherms to a one-site binding model. The figures were created with Origin 7 (OriginLab Corp., Northampton, MA, USA; https://www.originlab.com).

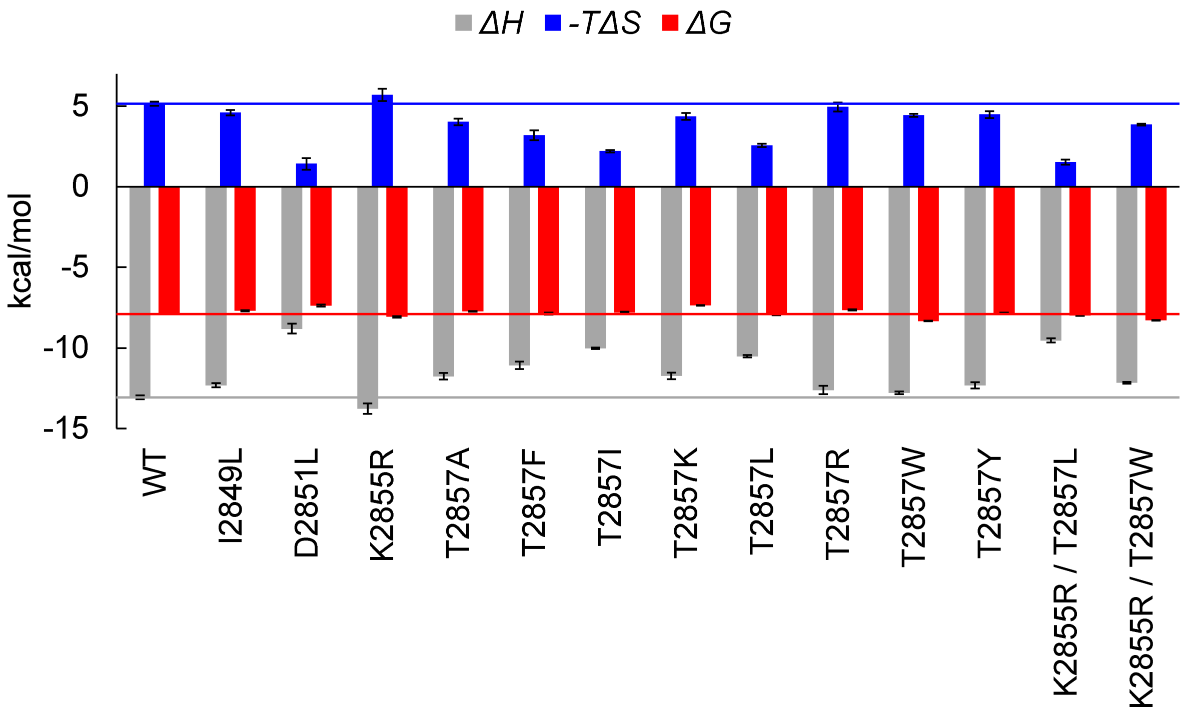

Supplementary Figure 6. Thermodynamic parameters for KIX binding of the wild type and mutants of the MLL TAD obtained by isothermal titration calorimetry. Thermodynamic parameters (enthalpy change ΔH, entropy change $-T\Delta S$, and free energy change ΔG) for KIX binding of each peptide are shown. The horizontal lines indicate the values of the wild-type MLL TAD. The figure was created with Microsoft Excel for Microsoft 365 (Microsoft Corp., Redmond, WA, USA; https://www.office.com).

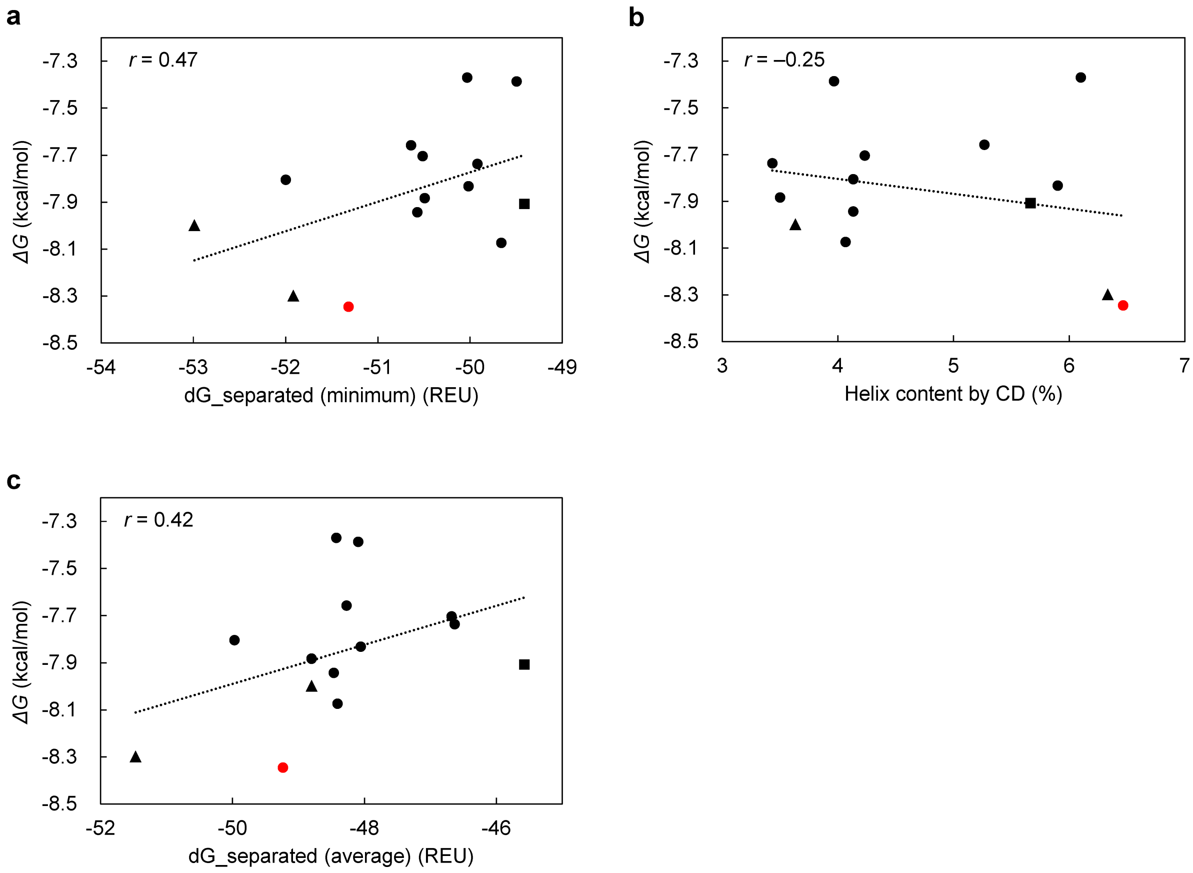

Supplementary Figure 7. Correlation with the KIX-binding affinity of the wild type and mutants of the MLL TAD peptide. The KIX-binding affinity was represented as the Gibbs free energy change (ΔG) upon binding (see Table 1). (**a**) Correlation between ΔG and the minimum dG_separated values (corresponding to binding energy between KIX and MLL TAD) predicted by Rosetta. The correlation coefficient, *r*, was 0.47. (**b**) Correlation between ΔG and helix contents of the MLL TAD peptides obtained from CD spectra ($r = -0.25$). (**c**) Correlation between ΔG and the average dG_separated values predicted by Rosetta ($r = 0.42$). In each panel, the T2857W mutant is shown by a red circle, the wild type by a black square, double mutants by black triangles, and other mutants by black circles. The figures were created with Microsoft Excel for Microsoft 365 (Microsoft Corp., Redmond, WA, USA; https://www.office.com).

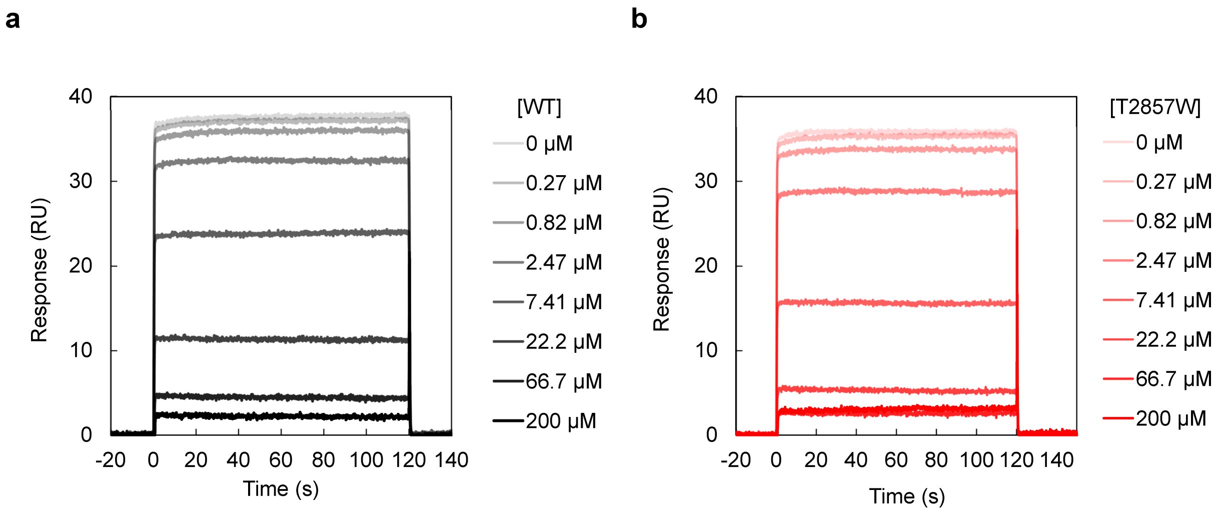

Supplementary Figure 8. Surface plasmon resonance sensorgrams of KIX binding to the immobilized MLL TAD in the presence of an inhibitor. The wild type (WT) (**a**) and T2857W mutant of the MLL TAD peptide (**b**) were used as an inhibitor. The peptide concentrations are shown. The KIX concentration was 5 μM. Panel (**b**) is the same as Fig. 5c and is shown for comparison. The figures were created with Microsoft Excel for Microsoft 365 (Microsoft Corp., Redmond, WA, USA; https://www.office.com).

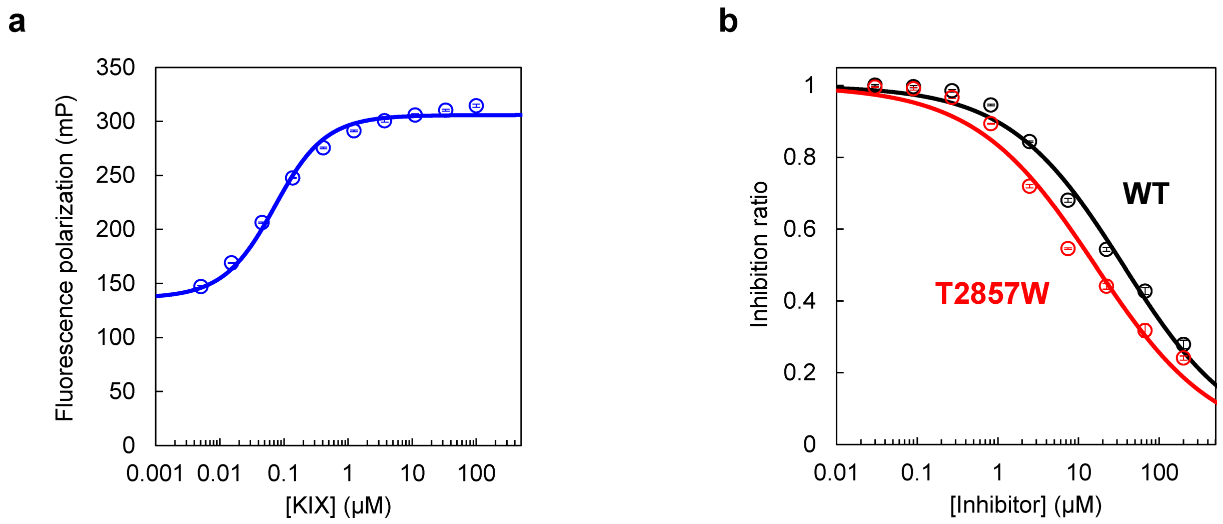

Supplementary Figure 9. Competitive inhibition assay of the MLL TAD peptides against the c-Myb–KIX interaction using fluorescence polarization. (**a**) The titration curve of the KIX binding to the FITC-labeled c-Myb TAD peptide (FITC-Myb). The continuous line was obtained by fitting to Eq. (6). The K_d was 0.059 ± 0.001μM. (**b**) Inhibition of KIX binding to FITC-Myb by an increasing concentration of an inhibitor (the wild type [WT] and T2857W mutant of the MLL TAD peptide). The IC_{50} value was 36 ± 1 μM for WT and 16 ± 1 μM for the T2857W mutant. The mean and standard error of triplicate measurements are shown. The figures were created with Microsoft Excel for Microsoft 365 (Microsoft Corp., Redmond, WA, USA; https://www.office.com).
